# Mental Health and Experiences of Anti‐Semitism in 2nd and 3rd Offspring Generation of Holocaust Survivors From Israel, Germany, and the USA

**DOI:** 10.1002/ijop.70053

**Published:** 2025-05-08

**Authors:** Yuriy Nesterko, Freya Specht, Nadine Stammel, Laura Nohr, Maria Böttche

**Affiliations:** ^1^ Department for Clinical Psychological Intervention Freie Universität Berlin Berlin Germany; ^2^ Department for Traumatic Stress and Transcultural Studies Center ÜBERLEBEN Berlin Germany; ^3^ Department for Medical Psychology and Medical Sociology University of Leipzig Medical Center Leipzig Germany

**Keywords:** perceived discrimination, psychological distress, Shoah, trauma

## Abstract

The existing and rising anti‐Semitism is a risk factor for the mental health of Jewish people worldwide. This study examines possible associations between anti‐Semitism and mental health in offspring/children (OHS) and grandchildren (GHS) of Holocaust survivors through cross‐country comparisons. A total of *n =* 248 OHS and *n =* 240 GHS from Israel, Germany, and the US completed a cross‐sectional online survey on experiences of anti‐Semitism, psychological distress, and posttraumatic stress symptoms, offered in English, German, and Hebrew. Psychological distress was significantly higher among participants from Germany vs. Israel and the US. Significant differences in experiences of anti‐Semitism were found between the generations, with higher rates in GHS. Experiences of anti‐Semitism were found to be associated with a higher risk for psychological distress and probable posttraumatic stress disorder (PTSD). The study emphasises the severe psychological stress being associated with experiences of anti‐Semitism among OHS and GHS across different countries of origin. Given the rise in anti‐Semitism since October 7, 2023 onwards, the findings are a warning and a clear impetus for political authorities as well as civil society to strengthen efforts for better healthcare and protecting Jewish life worldwide.

**Abbreviations**
AOR: adjusted odds ratio
CoO: country of origin
GHS: grandchildren of Holocaust survivors
OHS: offspring (children) of Holocaust survivors
PTSD: posttraumatic stress disorder
USA: United States of America

## 1 Introduction

Research on the Holocaust as one of the most significant collective traumas in modern history and its impact on the survivors, as well as their children/offspring (OHS) and grandchildren (GHS), has shaped the understanding of trauma and its consequences during the last decades (Danieli et al. 2017; Lehrner and Yehuda 2018; Kellermann 2023; Greenfeld et al. 2022). Such research has provided evidence of the manifold short‐ and long‐term consequences of traumatic experiences from an individual, community, and global perspective (Kellermann 2023). Research on the psychological consequences of the Holocaust began in the late 1960s and early 1970s (Greenfeld et al. 2022). In the 1980s, research on secondary traumatization, as well as the possible transmission of trauma to subsequent generations in the context of the Holocaust, increased in scope (Danieli et al. 2017). Since then, further studies have ensued, with various foci, methodologies, and levels of complexity (Kellermann 2023).

Current evidence on the Holocaust‐related trauma in survivors and their offspring is rather mixed. While meta‐analytic findings have suggested no transmission to the children or grandchildren of Holocaust survivors in non‐clinical samples (Sagi‐Schwartz et al. 2008; van IJzendoorn et al. 2003), more recent reviews have indicated a higher prevalence of mental health symptoms in OHS compared with controls (Payne and Berle 2021) and reported associations between parental mental health and OHS mental health (Dashorst et al. 2019). Moreover, studies have almost consistently demonstrated that OHS report higher levels of secondary traumatization compared to control offspring with no direct relatives being Holocaust survivors (Hoffman and Shrira 2019; Greenblatt‐Kimron et al. 2023), with slightly increased levels of symptoms of posttraumatic stress disorder (PTSD) in GHS (Payne and Berle 2021). However, there are also reports of high resilience in OHS and GHS (Lehrner and Yehuda 2018). From today's perspective, the association between the traumatization of parents and possible effects on their offspring is complex (Greenblatt‐Kimron et al. 2023), with no clear evidence‐based determinants of intergenerational transmission (Kellermann 2023). One possible determinant might be the next generations' own reflection on and identification with their Jewish heritage, e.g., ethnic and/or religious affiliation, cultural identity, experiences with anti‐Semitism, context of socialisation, and migrant status. For instance, a study on 98 adults with relatives who had either been killed during the Holocaust or survived the Holocaust found that stronger Jewish identity was associated with greater historical loss awareness (Johns et al. 2022). Furthermore, the manner of communication about the Holocaust among survivors impacts the next generations' perception of and reactions to historical trauma, as well as the mental well‐being of the offspring (Johns et al. 2022; Shrira 2016). For example, the children and grandchildren of Holocaust survivors who talked about their experiences in an impulsive and flooding manner, as well as those who remained silent or exclusively used indirect and guilt‐inducing communication, were found to have poorer mental health. Open and clear communication about the Holocaust, by contrast, was shown to positively impact mental health in the OHS and GHS generations (Johns et al. 2022; Shrira 2016). Furthermore, recent research has also shown positive associations between PTSD symptoms in Holocaust survivors and Holocaust centrality (i.e., the extent to which a traumatic event serves as a reference point for interpreting everyday experiences according to Berntsen and Rubin 2006) in subsequent generations (Greenblatt‐Kimron et al. 2021). Additionally, Holocaust centrality and symptoms of anxiety have been found to be positively associated with heightened sensitivity to terror threat in both OHS and GHS (Greenblatt‐Kimron et al. 2024). In a recently published study on OHS and GHS in Israel before and after the terrorist attack on October 7, Shrira et al. (2025) found increased probable PTSD rates among Holocaust descendants compared to controls without Holocaust backgrounds following the attack and during the ongoing war. This was particularly pronounced among OHS and GHS who reported probable PTSD in their parents or grandparents.

Since October 7, 2023, anti‐Semitic incidents and threats have risen globally. In Germany, for example, 994 incidents were recorded in the following month alone, averaging 29 incidents per day and marking a 320% increase compared to the same period in 2022 (RIAS 2023). In the US, the Anti‐Defamation League documented 5204 incidents between October 7 and the end of 2023 (ADL 2024). But even before, the European Union Agency for Fundamental Rights (FRA) (2019) found that a large majority of European Jewish respondents considered anti‐Semitic harassment, discrimination, and violence to be a problem in their respective home country (85%) and believed that anti‐Semitism had increased since 2003 (89%). In 2021, the Amadeu Antonio Foundation (2022) recorded the highest number of anti‐Semitic incidents in Germany since 2008, and the ADL reported a monthly increase of incidents in the first half of 2023 (ADL 2024).

Despite the growing concern over rising anti‐Semitism, only a few studies have examined its associations with mental health in general Jewish populations and, especially within the population of OHS and GHS in different countries. In one quantitative study, in which the majority of respondents were from the US, levels of anti‐Semitism were significantly associated with depression, survivor guilt proneness, neuroticism, and self‐hate (Kosdon et al. 2021). In addition, there are general findings on the negative correlation between discrimination, experiences of violence, experiences of threat, and psychological well‐being (Gerth et al. 2024). Regarding the Jewish population in Germany, Shani et al. (2025) identified associations between experiences of anti‐Semitism, along with perceived potential anti‐Semitic threats, and poorer mental health, increased withdrawal behaviour, and more pronounced symptoms of anxiety and depression. More broadly, perceived discrimination—defined as an individual's belief or perception that they have been treated unfairly or unfavourably due to a particular characteristic, such as race, gender, age, or other social identity traits (Krieger et al. 2005)—has been linked with multiple adverse health outcomes, including mental disorders, self‐rated health, cardiovascular health, and mortality (Barnes et al. 2008; Guyll et al. 2001; Karlsen and Nazroo 2002; Nesterko et al. 2018; Schunck et al. 2014; Troxel et al. 2003). The negative impact on physical health appears to be entirely mediated by poorer mental health as a result of experiences of discrimination (Schunck et al. 2014). The majority of studies examining mental health have focused on the impact of perceived discrimination on depression and anxiety (Wei et al. 2012).

In sum, the existing studies examining the mental health of the descendants of Holocaust survivors were conducted in Israel and the US, and yielded inconsistent findings regarding the descendants' mental health. To the best of our knowledge, no previous study has elaborated on the impact of anti‐Semitism among OHS and GHS before October 7 and based on cross‐country comparisons. The objective of the present study was therefore to examine sociodemographic and survivorship‐related characteristics in OHS and GHS from different countries of origin (Israel, Germany, and the US), with a particular focus on experiences of anti‐Semitism and its impact on mental health. We hypothesised that participants who grew up in Germany would report more experiences of anti‐Semitism compared to their peers in the US and Israel. We furthermore hypothesised that reported experiences of anti‐Semitism would be associated with symptoms of PTSD and psychological distress. However, it is important to emphasise that these hypotheses were formulated in an exploratory manner, and should thus be understood as a first approach towards a better understanding of potential differences across different generations and countries of origin of OHS and GHS.

## 2 Methods

We conducted a cross‐sectional online survey using Unipark (Questback GmbH 2021) from November 2021 to April 2022, targeting OHS and GHS growing up in Israel, Germany, and the US. The ethics committee of the Freie Universität Berlin approved the study (040/2021).

### 2.1 Participants

To be eligible to take part in the survey, participants had to be at least 18 years of age and to identify as a child or grandchild of Holocaust survivors growing up in Israel, Germany, or the US. The survey was accessible via a link that was distributed through social media posts (e.g., Facebook), local welfare organisations, NGOs, personal contacts, and mailing lists and newsletters of Jewish communities and/or local synagogues. Potential participants were informed about the voluntary and anonymous nature of study participation. No financial compensation was offered.

A total of 948 individuals viewed the page with the study information (comprising the study aims and duration as well as information on privacy and data protection), of whom 747 individuals proceeded to the informed consent page. In total, 691 participants provided informed consent to participate and continued to fill out the questionnaires (*n* = 4 did not give their active consent). Over the course of the survey, 167 participants dropped out, and 9 participants stated that they were over 77 years old, meaning that they could be considered as survivors and were therefore excluded from the analysis. Moreover, 26 participants who participated in the survey did not grow up in one of the three specified countries. Thus, *N* = 488 participants who completed the survey were included in the final sample. The mean length of time to complete the survey was 14 min.

### 2.2 Measures

The questionnaires and sociodemographic characteristics were administered in the respective languages of the three countries (English, German, and Hebrew). When no translations were available, a three‐step approach was used to obtain the final translation. First, two professional translators independently translated and back‐translated the questionnaires and sociodemographic questions for the respective language. Second, the two translators discussed discrepancies between the translations and agreed on the best possible translation. Third, the last author discussed potentially ambiguous items with the translators to eliminate discrepancies.

Participants provided information regarding their age, gender, country of origin (i.e., growing up in this country), migration status (yes/no), educational level, marital status, identifying as belonging to the OHS or GHS, number of family members who were Jewish Holocaust survivors (parents/grandparents), knowledge about family members' Holocaust experiences (How much do you know about your family members' Holocaust experiences? (1) nothing at all–(10) very much), and experiences of anti‐Semitism/exclusion due to Jewish origin (Have you ever felt excluded or invalidated because of your Jewish origin?). For later analyses in terms of better interpretation of possible associations, the variable was dichotomized into experiences of anti‐Semitism—(2) sometimes, (3) often, or (4) all the time, and no experiences of anti‐Semitism—(1) never.

To assess psychological distress, the 10‐item self‐report Kessler Psychological Distress Scale (K10; Kessler et al. 2002) was used. Items are rated on a 5‐point Likert scale (1 = none of the time to 5 = all of the time), yielding a sum score ranging from 10 to 50. A score at or above the cut‐off score of 25 indicates at least moderate psychological distress. For better interpretation of possible associations, the variable was dichotomised into psychological distress and no psychological distress. The psychometric properties of the scale have been established (Andersen et al. 2011; Giesinger et al. 2008; Peixoto et al. 2021). The scale was already available in English, Hebrew, and German. Cronbach's alpha in the present study was *α* = 0.93 (0.92–0.94 for the different language versions).

The Primary Care PTSD Screen for DSM‐5 (PC‐PTSD‐5; Prins et al. 2015) was developed to identify individuals with probable PTSD. The measure begins with an item assessing lifetime exposure to potential traumatic events. If the respondent indicates trauma exposure, five further items (yes/no) are completed, yielding a sum score ranging from 0 to 5. In the present study, participants with a sum score ≥ 4 were classified as having probable PTSD as recommended by Prins et al. (2015). The scale was already available in English and German (Schäfer and Schulze 2010) and was translated into Hebrew for the purpose of the present study. Cronbach's alpha in the present study was *α* = 0.74 (0.71–0.76 for the different language versions).

### 2.3 Statistical Analyses

The analysis was performed on complete data, so no missing values had to be imputed. Descriptive statistical characteristics (frequencies and proportions) were used to describe the sample. Differences in demographics between the OHS and GHS were investigated using Chi‐square tests (*χ* ^2^) for nominal and categorical variables and t‐tests for continuous variables. A *p*‐value of < 0.05 was considered to be statistically significant. Logistic regression models were performed, and adjusted odds ratios (AOR) were analysed to quantify associations between (1) socio‐demographics, (2) familial Holocaust‐related characteristics, and (3) experiences of anti‐Semitism and mental health outcomes (psychological distress and PTSD). The analyses were performed using IBM SPSS version 29 for Windows.

## 3 Results

### 3.1 Sample Characteristics

Of the total sample (*N* = 488), 336 (68.9%) participants were female, 151 (30.9%) were male, and one participant (0.2%) identified as gender diverse. The age ranged from 18 to 76 years, with a mean age of 52.8 years (SD = 15.1). The largest proportion of participants was originally from Israel (*n* = 310, 63.5%), followed by the US (*n* = 113, 23.2%) and Germany (*n* = 65, 13.3%). The majority were in a relationship or married (*n* = 344, 70.5%) and held a university degree (*n* = 355, 72.7%). In total, 248 participants (50.8%) stated that they were OHS. OHS and GHS differed in terms of age, country of origin, migration status, knowledge about Holocaust experiences of their family members, and experiences of anti‐Semitism. For detailed information, see Table 1.

**TABLE 1:** Sociodemographic and survivorship‐related characteristics stratified by OHS and GHS.

	OHS, *n* = 248	GHS, *n* = 240	*p* (*t*/X^2^)	Total sample, *N* = 488
Age			**< 0.001**	
*M*/SD/range	63.9/7.7/40–76	41.3/12.0/18–75		52.8/15.1/18–76
Gender			**0.**547	
Female	169 (68.1%)	167 (69.6%)		336 (68.9%)
Male	79 (31.9%)	72 (30%)		151 (30.9%)
Other	—	1 (0.4%)		1 (0.2%)
Country of origin			**0.016**	
Israel	157 (63.3%)	153 (63.7%)		310 (63.5%)
Germany	24 (9.7%)	41 (17.1%)		65 (13.3%)
USA	67 (27%)	46 (19.2%)		113 (23.2%)
Migrated			**< 0.001**	
Yes	20 (8.1%)	75 (31.3%)		95 (19.5%)
No	228 (91.9%)	165 (68.8%)		393 (80.5%)
University degree			0.271	
Yes	175 (70.6%)	180 (75%)		355 (72.7%)
No	73 (29.4%)	60 (25%)		133 (27.3%)
Partnership/marriage			0.068	
Yes	184 (74.2%)	160 (66.7%)		344 (70.5%)
No	64 (25.8%)	80 (33.3%)		144 (29.5%)
Number of survivors			0.299	
*M*/SD/range	2.3/1.4/1–6	2.4/1.1/1–6^a^		2.4/1.3/1–6
Knowledge about family members' Holocaust experiences			**0.007**	
*M*/SD/range	7.1/2.1/2–10	6.6/2.3/1–10		6.8/2.2/1–10

^a^ *N* = 238.

Bold values highlights the significant effects.

### 3.2 PTSD and Psychological Distress in OHS and GHS

**FIGURE 1:**
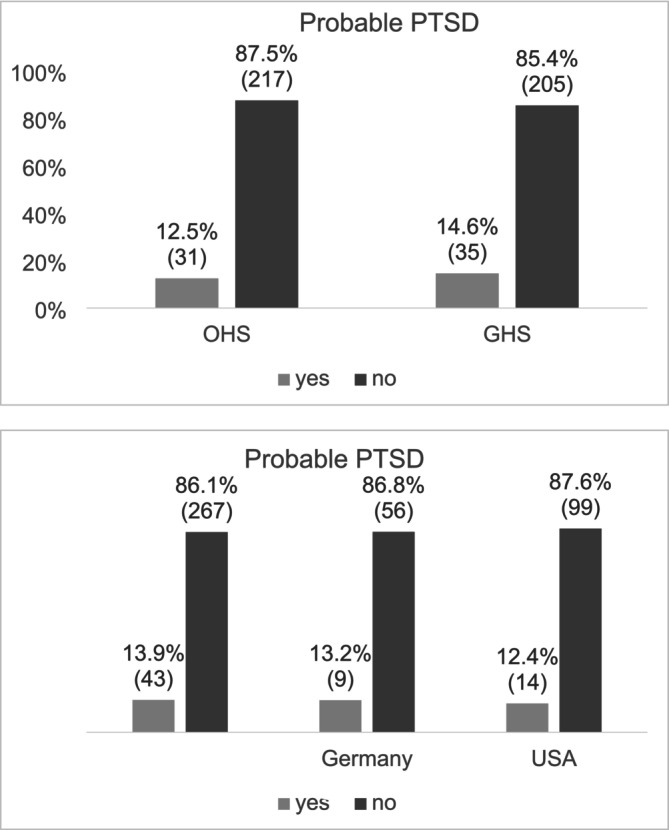
Presence of probable PTSD by generation and country of origin. *N* = 488. Numbers in parenthesis indicate the number of individuals in each category.

Prevalence rates of probable PTSD and psychological distress were calculated for all participants and stratified by country of origin as well as by generation status. A total of 66 participants (13.5%) were classified as having probable PTSD, with no significant differences between the generations, *χ* ^2^(1) = 0.453, *p* = 0.501, or countries of origin, *χ* ^2^(2) = 0.162, *p* = 0.922 (Figure 1).

**FIGURE 2:**
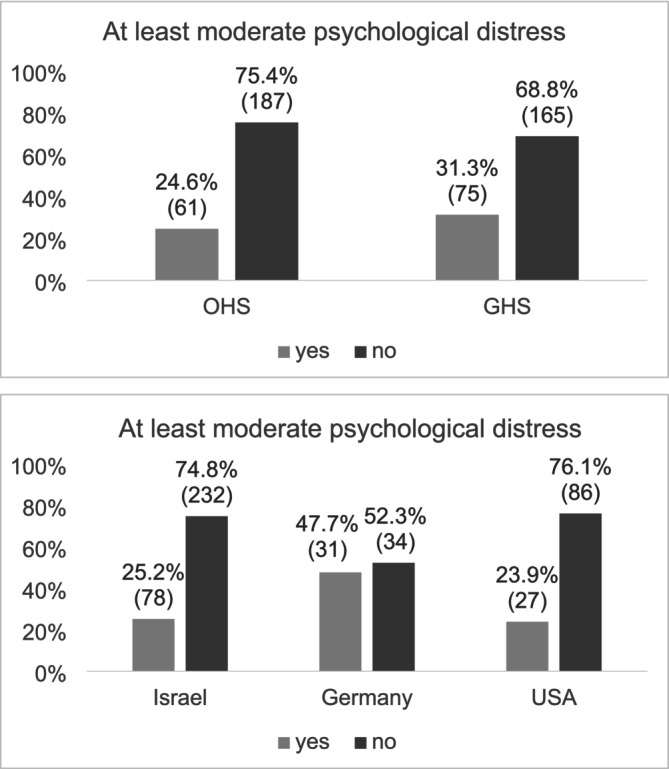
Moderate psychological distress by generation and country of origin. *N* = 488. Numbers in parentheses indicate the number of individuals in each category.

At least moderate psychological distress was found in 136 participants (27.9%), with no significant differences between the generations, *χ* ^2^(1) = 2.686, *p* = 0.101 (Figure 2). Significant differences were found between countries of origin, *χ* ^2^(2) = 14.725, *p* < 0.001, with a higher proportion of participants from Germany (47.7%) exhibiting at least moderate psychological distress compared to those from Israel (25.2%) and the United States (23.9%).

### 3.3 Experiences of anti‐Semitism in OHS and GHS

**FIGURE 3:**
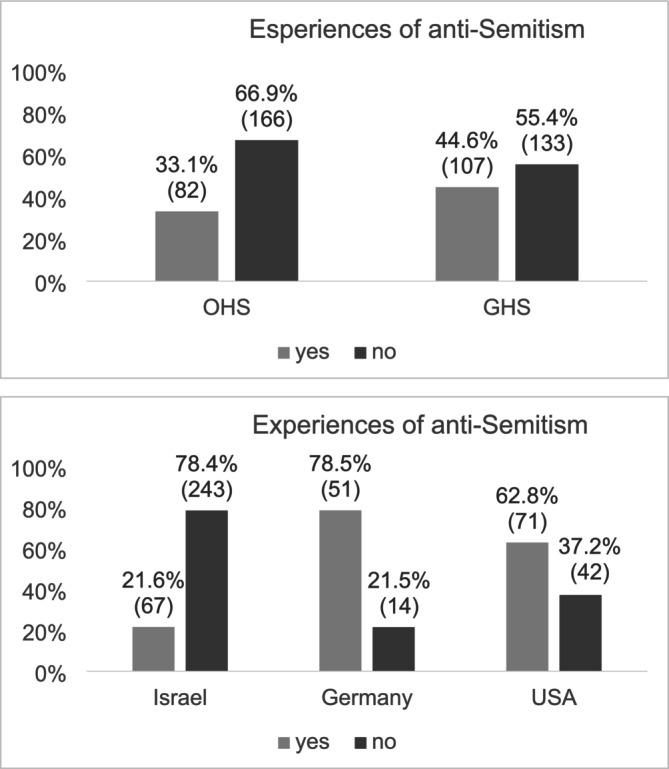
Experiences of anti‐Semitism by generation and by country of origin. *N* = 488. Numbers in parentheses indicate the number of individuals in each category.

Reported experiences of anti‐Semitism were calculated for participants in total and stratified by country of origin as well as by generation status. Overall, 189 (38.7%) participants reported having experienced anti‐Semitism at least sometimes. Significant differences were found between the generations, *χ* ^2^(1) = 6.820, *p* = 0.009, revealing more often experiences of anti‐Semitism among GHS compared to the OHS (44.6% vs. 33.1%; Figure 3). Furthermore, significant differences emerged between countries of origin, *χ* ^2^(2) = 109.179, *p* < 0.001, with the most experiences of anti‐Semitism reported by participants originating from Germany (78.5%), followed by the US (62.8%), and lastly Israel (21.6%) (Figure 3).

### 3.4 Prediction of Psychological Distress and PTSD in OHS and GHS

Table 2 shows the results of the two multivariable logistic regression analyses to determine the associations between age, gender, partnership/marriage status, country of origin, migration status, number of survivors, knowledge about family members' Holocaust experiences, and experiences of anti‐Semitism on the likelihood of psychological distress and probable PTSD in OHS and GHS.

**TABLE 2:** Logistic regression analyses predicting psychological distress and PTSD in OHS and GHS.

	Psychological distress			PTSD		
Predictors	AOR	95% CI	*p*	AOR	95% CI	*p*
Age	**0.964**	**0.942–0.988**	**0.003**	0.990	0.962–1.018	0.469
Gender^a^	**0.520**	**0.316–0.857**	**0.010**	0.719	0.388–1.331	0.294
University degree^b^	1.288	0.778–2.132	0.325	0.848	0.465–1.547	0.591
Partnership/marriage^b^	**0.437**	0.**277–0.687**	**< 0.001**	0.782	0.438–1.394	0.404
CoO Israel^b^	1.454	0.799–2.646	0.221	1.843	0.868–3.910	0.111
CoO Germany^b^	**2.449**	**1.197–5.010**	**0.014**	0.926	0.361–2.376	0.874
Migrated^b^	1.366	0.766–2.437	0.291	0.885	0.415–1.887	0.751
Number of survivors	1.018	0.856–1.212	0.837	1.026	0.827–1.274	0.813
Generation^c^	**0.467**	**0.231–0.943**	**0.034**	0.816	0.354–1.884	0.635
Knowledge about family members' Holocaust experience	1.027	0.927–1.138	0.615	1.007	0.888–1.143	0.908
Experiences of anti‐Semitism^b^	**2.256**	**1.378–3.693**	0.**001**	**3.194**	**1.712–5.959**	**< 0.001**
**Model fit indices:**						
*χ* ^**2**^ **/df/*p***	**66.311/11/< 0.001**			17.855/11/0.085		
**−2 Log‐Likelihood**	**509.899**			364.573		
**Nagelkerke's *R*** ^**2**^	**0.184**			0.066		
**Cox and Snell *R*** ^**2**^	**0.128**			0.036		

*Note*: AOR = adjusted odds ratio; CoO = country of origin.

^a^ Female = 1, male = 2.

^b^ yes = 1, no = 0 (with the reference category being US as the country of origin).

^c^ OHS = 1, GHS = 2.

Bold values highlights the significant effects.

The logistic regression model predicting psychological distress was statistically significant, *χ*2(11) = 66.311, *p* < 0.001. The model explained 18.4% (Nagelkerke *R* ^2^) of the variance in psychological distress and correctly classified 72% of cases. Psychological distress was more likely in younger participants compared to older participants (AOR = 0.96, 95% CI [0.94, 0.99]), in participants identifying as female compared to those identifying as male (AOR = 0.52, 95% CI [0.32, 0.86]), and in single participants compared to those in a partnership or marriage (AOR = 0.44, 95% CI [0.28, 0.69]). Participants who originated from Germany were more than twice as likely to suffer from psychological distress compared to those who originated from the US (AOR = 2.44, 95% CI [1.1895.01]). Moreover, OHS were more likely to suffer from psychological distress compared to GHS (AOR = 0.47, 95% CI [0.23, 0.94]). Participants reporting experiences of anti‐Semitism showed a twofold higher risk of experiencing psychological distress (AOR = 2.25, 95% CI [1.37, 3.69]). Having a university degree, Israel as the country of origin (compared to the US), migration status, number of survivors within the family, and knowledge about family members' Holocaust experience were not significantly associated with psychological distress. The effects reported above are associations that were controlled for the confounding influence of other predictors of the model.

The logistic regression model predicting probable PTSD and its predictor variables was not statistically significant, *χ* ^2^(11) = 17.855, *p* < 0.085, but did indicate that participants who reported experiences of anti‐Semitism were three times more likely to suffer from probable PTSD than those who did not report experiences of anti‐Semitism (AOR = 3.19, 95% CI [1.71, 5.96]).

## 4 Discussion

The present study analysed psychological distress, probable PTSD, and experiences of anti‐Semitism among OHS and GHS originating from Israel, Germany, and the US. At the descriptive level, OHS and GHS did not significantly differ regarding levels of psychological distress and probable PTSD. No differences in probable PTSD were found between countries of origin, but participants from Germany reported higher rates of psychological distress compared to those from Israel and the US. Regarding experiences of anti‐Semitism, GHS reported significantly more often anti‐Semitic experiences compared to OHS, and descendants from both generations growing up in Germany experienced the highest levels of anti‐Semitism compared to OHS and GHS from Israel and the US.

However, when investigating psychological distress, age, gender, marital status, country of origin, and generation status were found to be significantly associated factors indicating greater distress in younger participants, females (vs. males), single participants (vs. partnered participants), participants originating from Germany (vs. the US), and OHS (vs. GHS). No such associations were found for probable PTSD. Regarding the proposed hypotheses, we found that respondents from Germany reported more frequent experiences of anti‐Semitism compared to those from Israel and the US. Additionally, the negative association between experiences of anti‐Semitism and the mental health of those affected was demonstrated.

In the current study, 13.3% of respondents reported probable PTSD. In a study by Yehuda et al. (2008) involving 200 OHS, the PTSD prevalence was 19%. When compared to the general population of the three countries, the corresponding prevalences of PTSD are significantly lower. For example, the 1‐month prevalence of 1.5% in Germany (Maercker et al. 2018), a lifetime prevalence of 9% in Israel (Ben‐Ezra et al. 2018), and a lifetime prevalence of 3.4% in the US (Cloitre et al. 2019) suggest an increased risk of developing PTSD in the population of OHS and GHS. However, this should be interpreted with caution, as the current study relied on a basic, though well‐validated, questionnaire and the investigated sample is not representative.

It should also be noted that the data collection was finished nearly 2 years before the events of October 7, 2023. Recent studies conducted after the terrorist attack report substantially higher prevalence rates for PTSD and other mental disorders in the general Israeli population (Levi‐Belz et al. 2024; Palgi et al. 2024), as well as among Holocaust survivors and their descendants (Shrira et al. 2025). Levi‐Belz et al. (2024) found that the prevalence of probable PTSD in the general Israeli population nearly doubled, increasing from 16.2% before the attack to 29.8% 1 month after. Symptoms of generalised anxiety disorder and depression also showed significant increases, with direct exposure to the attack identified as a key risk factor for PTSD and depression. Palgi et al. (2024) further highlighted not only high levels of probable PTSD but also an increased subjective perception of being traumatised. Among Holocaust descendants, Shrira et al. (2025) found that probable PTSD rates increased disproportionately. While pre‐war PTSD rates were similar between Holocaust descendants and a comparison group (10.4% vs. 11.5%, respectively), post‐attack PTSD rates rose significantly more among Holocaust descendants (20.9% vs. 11.5%). Notably, those whose survivor parents or grandparents had probable PTSD were at an even greater risk of developing PTSD themselves. These findings suggest that Holocaust descendants may carry an increased vulnerability, particularly when exposed to new large‐scale traumatic events.

No significant association was found between the number of Holocaust survivors in the family and the mental health of the offspring. This finding contradicts the results of the review by Dashorst et al. (2019), who showed that offspring with two surviving Holocaust parents experienced greater mental health problems. However, our analyses did not differentiate between one or two Holocaust survivor parents, but instead included the number of all family members (i.e., potentially also grandparents on both sides, with a total range in this sample of 1–6) in the models. It is therefore feasible that the direct impact of one generation will no longer be evident when considering the next generation. The same applies with regard to knowledge about the Holocaust experiences within the family (e.g., experiences of parents or grandparents or both parents and grandparents). However, for example, Greenblatt‐Kimron et al. (2023) found evidence of secondary traumatization in both OHS and GHS populations in Israel, suggesting that trauma‐related distress can extend beyond direct parent–child transmission. Future qualitative research may offer a more nuanced understanding of this interplay.

The strongest associations for the prediction of psychological distress and probable PTSD were found for experiences of anti‐Semitism. Specifically, participants who reported experiences of anti‐Semitism were twice as likely to show at least moderate psychological distress and three times more likely to show probable PTSD when controlling for the other factors. These results support previous findings on ethnic discrimination or racism and its impact on mental health (Paradies et al. 2015), and they clearly underline the significant negative associations between anti‐Semitism and mental health and well‐being. In total, 189 participants (38.7%) reported experiences of anti‐Semitism, and such experiences were most prevalent among GHS and those originating from Germany. The latter finding echoes numerous reports of increased rates of anti‐Semitism in Germany over the past 10 years (e.g., Decker et al. 2023; Gerth et al. 2024; Shani et al. 2025). Unfortunately, no previous research has similarly examined experiences of anti‐Semitism and its impact on the mental health among OHS and GHS from Israel, Germany, and the US. Therefore, future research looking more closely at cross‐national comparisons in the descendants of Holocaust survivors is needed.

## 5 Limitations

Some limitations of the present study need to be considered. First, the sample is not representative for the descendants of Holocaust survivors, and a sampling bias cannot be ruled out due to the voluntary convenience sampling methods. As we did not include a control group of participants without relatives who survived the Holocaust, we are unable to determine the extent to which psychological distress and PTSD are specific to the descendants of Holocaust survivors. Second, the cross‐sectional nature of this study precludes causal inferences. Third, due to the short screening assessments used, e.g., measuring experiences of anti‐Semitism using only one question, we are unable to elaborate on the associations found in detail. Thus, future research is needed to assess anti‐Semitism and mental health symptoms more comprehensively. Lastly, the different sample sizes of the subgroups of OHS and GHS from the three different countries rather limit the ability to draw profound cross‐country comparisons. For example, from the relatively small German subsample, we cannot determine whether a more comprehensive sample would show the same level of psychological distress or whether we had reached a severely affected subgroup. Furthermore, it is important to recognise that anti‐Semitism is a complex phenomenon that requires deeper examination. This should not only involve comparisons between countries and generations, but also consider structural and context‐specific factors, such as the historical legacy of Jewish life in each country and the rise of right‐wing political ideologies of nationality. In addition, findings from surveys on anti‐Semitism may be subject to fluctuations and affected by public and social media discourse, such as those observed since October 7, 2023.

## 6 Conclusions

The aforementioned limitations notwithstanding, the present study has a number of strengths. In particular, the hitherto unique cross‐country comparative approach revealed specific differences between countries of origin within OHS and GHS. Moreover, we were able to provide first insights into the association of anti‐Semitism with psychological distress and PTSD in three different countries. In general, the rate of psychological distress in the present sample is rather high. While we are unable to conclude whether this is related to the Holocaust experiences of the respondents' parents and/or grandparents, we can state that the descendants of Holocaust survivors from different countries of origin constitute a risk group for developing mental disorders, regardless of whether this is due to historical trauma in their families, current exposure to anti‐Semitism, or other traumatic events. These findings of increased psychological symptoms contribute to the discussion on possible long‐term effects of the Holocaust and have implications for the broader context of survivors of catastrophes on a global level.

Furthermore, the findings on associations between experiences of anti‐Semitism and mental health emphasise the existing problem of discrimination against and threats to Jewish people, especially those in Germany. In addition to further in‐depth research into this phenomenon and its consequences, clear implications can be drawn for policymakers and civil society. In light of October 7, 2023, and the subsequent global rise in anti‐Semitism, there is an urgent need to raise awareness and resolutely oppose this specific form of aggression.

## Ethics Statement

The studies involving human participants were reviewed and approved by the ethics committee of the Freie Universität Berlin. The participants provided written informed consent to participate in this study.

## Consent

Informed consent was obtained from all subjects involved in the study.

## Conflicts of Interest

The authors declare no conflicts of interest.

## Data Availability

The detailed sociodemographic information of the dataset does not fully protect the anonymity of the respondents. For this reason, the entire dataset cannot be made publicly available. However, excerpts of the data on a higher aggregation level can be provided upon justified request to the corresponding author.
